# Lignin and Xylan as Interface Engineering Additives for Improved Environmental Durability of Sustainable Cellulose Nanopapers

**DOI:** 10.3390/ijms222312939

**Published:** 2021-11-29

**Authors:** Sergejs Beluns, Oskars Platnieks, Sergejs Gaidukovs, Olesja Starkova, Alisa Sabalina, Liga Grase, Vijay Kumar Thakur, Gerda Gaidukova

**Affiliations:** 1 Faculty of Materials Science and Applied Chemistry, Institute of Polymer Materials, Riga Technical University, P. Valdena 3/7, LV-1048 Riga, Latvia; Oskars.Platnieks_1@rtu.lv (O.P.); Vijay.Thakur@sruc.ac.uk (V.K.T.); 2 Institute for Mechanics of Materials, University of Latvia, Jelgavas 3, LV-1004 Riga, Latvia; olesja.starkova@lu.lv (O.S.); alisa.sabalina@edu.rtu.lv (A.S.); 3 Faculty of Materials Science and Applied Chemistry, Institute of Materials and Surface Engineering, Riga Technical University, P. Valdena 3, LV-1048 Riga, Latvia; liga.grase@rtu.lv; 4 Biorefining and Advanced Materials Research Center, SRUC, Edinburgh EH9 3JG, UK; 5 Department of Mechanical Engineering, School of Engineering, Shiv Nadar University, Greater Noida 201314, Uttar Pradesh, India; 6 School of Engineering, University of Petroleum & Energy Studies, Dehradun 248007, Uttarakhand, India; 7 Faculty of Materials Science and Applied Chemistry, Institute of Applied Chemistry, Riga Technical University, P. Valdena 3/7, LV-1048 Riga, Latvia; gerda.gaidukova@rtu.lv

**Keywords:** mechanical properties, sustainable, functional material, food packaging, photodegradation, moisture absorption

## Abstract

Cellulose materials and products are frequently affected by environmental factors such as light, temperature, and humidity. Simulated UV irradiation, heat, and moisture exposure were comprehensively used to characterize changes in cellulose nanopaper (NP) tensile properties. For the preparation of NP, high-purity cellulose from old, unused filter paper waste was used. Lignin and xylan were used as sustainable green interface engineering modifiers for NP due to their structural compatibility, low price, nontoxic nature, and abundance as a by-product of biomass processing, as well as their ability to protect cellulose fibers from UV irradiation. Nanofibrillated cellulose (NFC) suspension was obtained by microfluidizing cellulose suspension, and NP was produced by casting films from water suspensions. The use of filler from 1 to 30 wt% significantly altered NP properties. All nanopapers were tested for their sensitivity to water humidity, which reduced mechanical properties from 10 to 40% depending on the saturation level. Xylan addition showed a significant increase in the specific elastic modulus and specific strength by 1.4- and 2.8-fold, respectively. Xylan-containing NPs had remarkable resistance to UV irradiation, retaining 50 to 90% of their initial properties. Lignin-modified NPs resulted in a decreased mechanical performance due to the particle structure of the filler and the agglomeration process, but it was compensated by good property retention and enhanced elongation. The UV oxidation process of the NP interface was studied with UV-Vis and FTIR spectroscopy, which showed that the degradation of lignin and xylan preserves a cellulose fiber structure. Scanning electron microscopy images revealed the structural formation of the interface and supplemented understanding of UV aging impact on the surface and penetration depth in the cross-section. The ability to overcome premature aging in environmental factors can significantly benefit the wide adaption of NP in food packaging and functional applications.

## 1. Introduction

Cellulose nanopaper (CNP) is a prospective bio-based material made from nanofibrillated cellulose (NFC). The unique structure obtained from thin NFC films enables advanced applications such as biomedicine, smart and flexible electronics, and enhanced gas barrier membranes for filtering and food packaging purposes [1,2,3,4]. While various authors have focused mainly on the potential and properties of these materials, few studies examine their durability concerning environmental effects such as moisture and UV irradiation. Expensive nanoparticles or non-biodegradable additives have been mainly used to enhance NP properties, which unfortunately do not promote material competitiveness or meet sustainable technology goals [4,5,6]. The novel sustainable modification routes and bio-based modifiers to improve the durability of CNP are in high demand for the advancement of green chemistry [7,8].

Lignin and hemicellulose are green and abundant materials found naturally in plants; they are also produced as by-products in a variety of industries, for example, lignin is a major by-product of the paper industry [9,10]. They offer protection for cellulose in wood and plant structures by reducing the environmental impact of UV irradiation and microbial attacks. Some studies have characterized changes in the mechanical properties of NPs obtained with partial or full purification of NFC from lignin and hemicellulose [11,12,13,14]. However, these processes are often hard to control and repeat and depend on the source of cellulose [15,16]. Thus, there is a basis for exploring the controlled addition of these natural components rather than their removal from the NFC structure.

Hemicellulose can refer to various heteropolymers usually formed in different ratios in various plants and trees [17,18]. Xylan is one of the most abundant types of hemicellulose, and it is characterized by low oxygen permeability [19]. Thus, xylan can be used as an additive to protect material in a thermo-oxidative environment. Lignin is often regarded as a natural defense against UV radiation, as indicated by oxidation studies for papers, woods, and natural industrial fibers [20,21,22]. The oxidation starts with lignin, where β-O-4 linkage will likely undergo cleavage and produce free radicals [23]. After lignin oxidation, various oxidative reactions occur for cellulose hydroxyl groups at C(2), C(3), and C(6) atoms in a glucopyranose unit [24]. This leads to the formation of carbonyl groups as single ketones or conjugated diketones at C(2) and C(3) or aldehydes/carboxyls at C(6) [25,26,27]. If water participates in the oxidation process, glycosidic bond cleavage can occur at C(1) [24]. It has been reported that cellulose fibers without lignin degrade significantly faster, thus leading to a shorter material lifespan [21,28]. Due to xylan possessing a similar carbohydrate structure, it follows similar degradation routes.

The drawbacks of using cellulose and xylan are related to their hydrophilic structures with a high affinity for water [29]. The use of lignocellulose reduces water absorption [3,30]. Thus, lignin serves as an additional interface layer to protect the cellulose fibers from microbial attacks and subsequent biodegradation [31]. However, the use of lignin as a filler often results in reduced mechanical properties [32]. At the same time, some studies on micro and nanopapers have reported that enhanced mechanical properties can be achieved with the addition of hemicellulose [17,33]. This is explained by the branched hemicellulose structure possessing a lower molecular weight compared to cellulose fibrils. Thus, xylan (hemicellulose) effectively fills gaps, creates strong hydrogen bonding between cellulose fibrils, increases their entanglement, and develops a protective interface layer, benefiting the mechanical properties [34,35].

**Figure 1:**
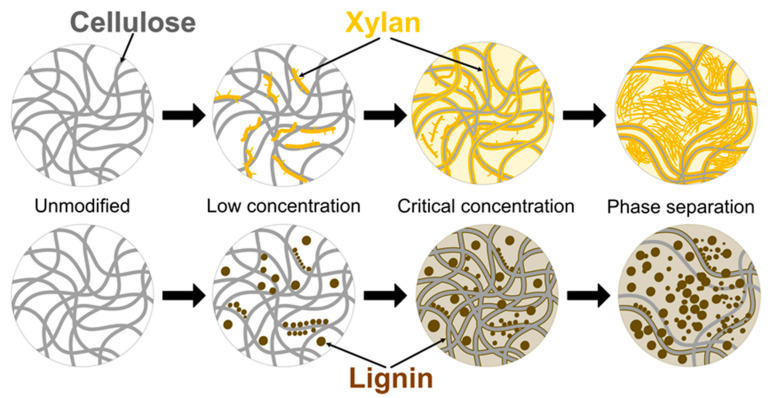
Schematic representation of the nanopapers structure.

Given the tremendous interest in NPs, we undertake in the present study an in-depth investigation of UV irradiation and moisture effects on the durability of modified NPs. Tensile properties were extensively tested before and after exposure to simulated environmental impacts, and UV-Vis, FTIR spectroscopies were used to supplement the analysis. We selected lignin and xylan as green, cheap, and nontoxic natural cellulose surface modifiers for NPs and examined their interface and interphase formation mechanisms to enhance properties. The analysis was supplemented with extensive SEM characterization that explored structural changes and showed aging damage to NPs. By targeting the interphase development observable at critical concentrations, the durability performance improvement technology is proposed. The proposed cellulose interface modification scheme is shown in Figure 1, and the modifier concentration is varied in a range from 1 to 30 wt%. With this research, the authors would like to address the limiting aspects of NP application, i.e., durability, and explore potential benefits added to structural integrity by mimicking plant structures, which are achieved by adding lignin and xylan.

## 2. Results and Discussion

### 2.1. UV Irradiation and Heat Effect on the Tensile Properties

**Figure 2:**
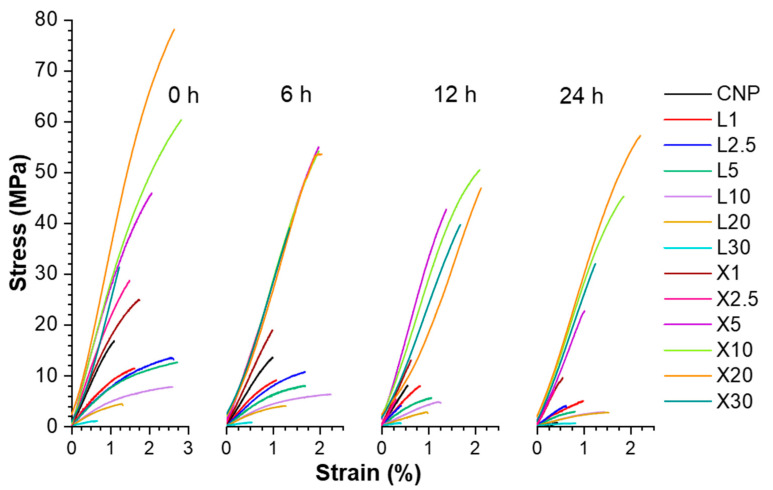
Representative stress–strain curves of NP for pristine and aged samples at different times of UV irradiation at 80 °C temperature. Lignin (L) and xylan (X) with their loading in wt%. E.g., X1 sample corresponds to NP with 1 wt% xylan and 99 wt% NFC loadings, L1 sample—lignin 1 wt% and NFC 99 wt%.

**Figure 3:**
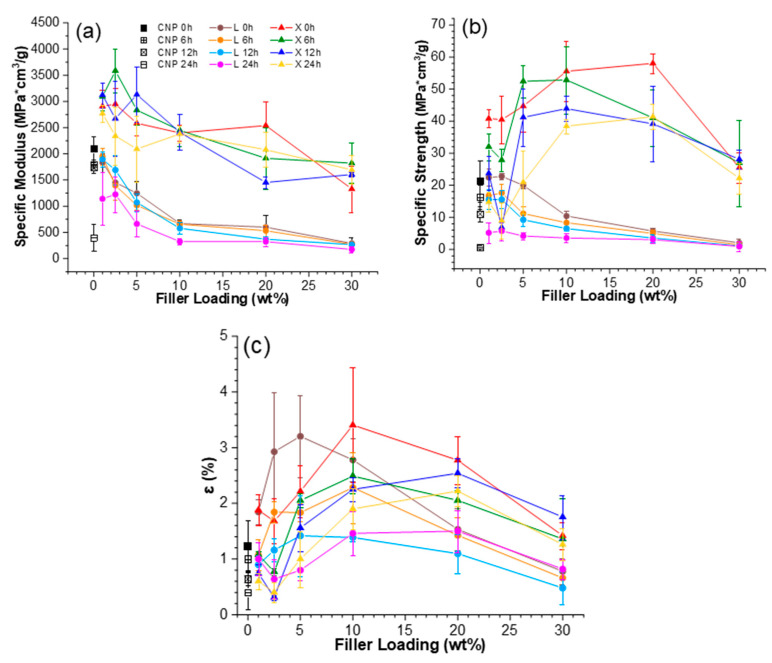
Tensile properties: (**a**) specific elastic modulus, (**b**) specific ultimate tensile strength and (**c**) elongation at break for nanopapers before and after exposure to intensive UV irradiation at 80 °C.

Figure 2 depicts sample stress–strain curves, and Figure 3 illustrates tensile properties for UV-irradiated samples. For the reference samples, the effects of lignin and xylan modifiers can be examined for their tensile properties, which indicate that the addition of xylan enhances all tensile properties, while lignin reduces the elastic modulus and tensile strength but increases the elongation at break values (Figure 3).

The addition of xylan to CNP showed an increase in NPs properties even with high loading up to 20 wt% for the specific elastic modulus and specific tensile strength, achieving up to 1.4-fold and 2.8-fold improvements, respectively. Elongation values reach a maximum value at 10 wt% xylan loading, showing a 2.8-fold improvement compared to CNP. In comparison, lignin addition resulted in a 2.6-fold increase in the elongation values, while specific elastic modulus exhibited a 1.1 to 3.6-fold decrease for lignin content up to 10 wt%. Specific tensile strength showed similar values to CNP with loadings up to 5 wt% lignin, but further addition resulted in almost a 2-fold decrease.

At higher lignin loadings, samples were fragile and showed an even further drop in properties. It was observed that modifier loading of 10 wt% corresponds to the xylan critical threshold concentrations for the developed interface structure. It could be explained that the biopolymer phase was segregated at higher loadings, while a single highly enhanced interface was produced with 2.5 wt% of lignin and 10 wt% of xylan modifier concentrations. For example, adding 2.5–10 wt% of xylan could result in complete cellulose surface modification, i.e., coating.

Berglund et al. reported that hemicelluloses could control cellulose fibril aggregation and complement interface with a combination of rigid and flexible interactions [36]. While at high loadings, segregated xylan biomolecules can form separate bulk interphase characterized by flexible molecular chains. Filler concentration at 10 wt% reaches critical threshold content values for a noticeable shift in different structural interactions. This is expressed as an almost linear increase of 2.5 to 10 wt% loadings for several affected compositions.

Kontturi et al. compared NFC NPs to bacterial cellulose (BC) NPs and found that their only difference was the presence of hemicellulose in the interface between the fibrils of NFC and no hemicellulose for BC [37]. The authors reported that NFC NPs had higher mechanical properties and concluded that hemicellulose significantly improves the interface adhesion between individual fibrils. As xylan is a type of hemicellulose with a more branched structure, it can form more hydrogen bonds on the interface, acting as an efficient glue and improving the overall mechanical properties. It has also been reported that xylan NPs have much lower mechanical properties than cellulose NPs [38,39]. Thus, at higher loadings such as the X30 sample, the cellulose fibril network is disrupted significantly, reducing mechanical properties. Similar observations about hemicellulose interface “glue” properties have been reported by other authors [40,41]. In comparison, the lignin remains as particles, forming the segregated interphase in the NP films. The distorted NFC mesh networks resulted in a decreased interface adhesion and mechanical performance of those lignin NP samples.

**Figure 4:**
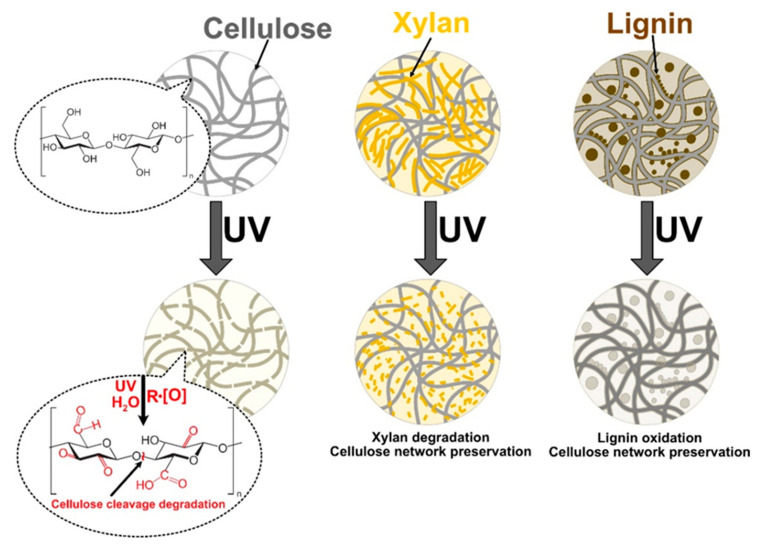
Visual representation of the oxidation process under UV ultraviolet light irradiation.

UV-irradiated CNP showed a catastrophic decrease in properties, and after 24 h of UV exposure, its properties were the lowest of any NP. It has been reported in the literature that cellulose is very unstable in UV light without stabilization additives [24,42,43]. The aged xylan NP samples demonstrated a shift to lower filler loadings that achieved the best properties. For xylan NPs, the best values after aging were for loadings from 5 to 20 wt%. Lignin-modified NPs showed a slight increase in elongation values and decreased elastic modulus and tensile strength compared to CNP. In addition, it was also observed that xylan loadings up to 5 wt% benefited from exposure to UV, resulting in the stiffening of NP. Figure 4 shows a proposed visual schematic illustration of the oxidation process at the interface of the obtained NPs. The illustration is used to complement the following discussion and UV-vis section.

The increase in the elastic modulus was observed for an exposure time of 6 h and partly for 12 h. This could be directly related to thermal oxidation and degradation processes, which are supported by a visible decrease in elongation values. Thus, smaller, more rigid sections are formed. As xylan fills gaps between cellulose fibrils, adsorbs, and protectively coats their interface (Figure 4), the aging process shifts more from volume to the interface and enhances the materials’ durability [44]. In addition, it is known that more branched and lower molecular weight biomolecules degrade before cellulose [45,46]. A significant property decrease was obtained for lignin NP after 24 h of UV exposure, while a milder effect was obtained after 12 h. We observed that after 12 h irradiation, a critical time is reached for the thermal oxidation process inhibition in modified nanopaper. At 24 h, the modifiers do not fully shield the cellulose fibers from UV light; then, the cellulose degrades intensively. It is observed as a drop in stiffness and strength. The xylan NP showed a remarkable ability to retain most of its properties after UV irradiation, which is related to the stabilization effect of modified NP.

**Figure 5:**
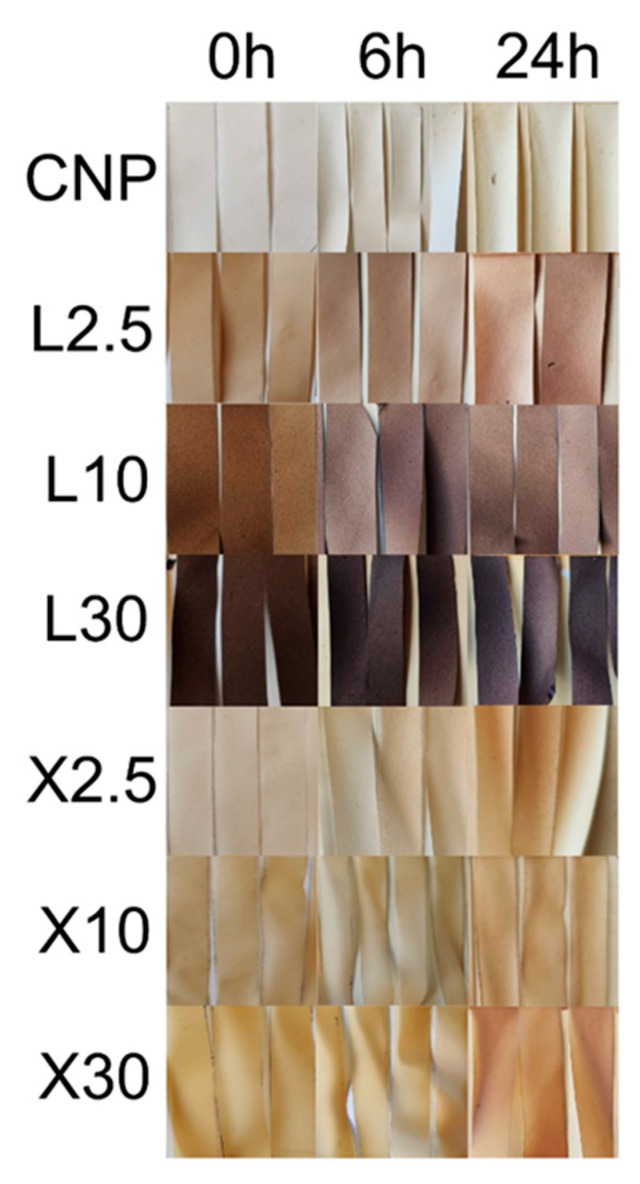
The visual appearance of NP before and after exposure to intensive UV irradiation at 80 °C.

The changes were also visual (Figure 5) as NP samples with longer exposure times had their color shift to a darker brown than reference samples. The color shift was very pronounced for xylan NP and correlated with higher xylan content. The high intensity of UV radiation oxidized oxygen to ozone, which assisted in the sample oxidation process. The oxidation process creates radicals that can induce chain scission, thus reducing the components’ molecular weight and mechanical strength [47]. In addition, heat exposure has been reported to induce chain relaxation, promoting cellulose crystallization and the reformation of hydrogen bonds [29]. Thus, two opposing effects take place during the sample’s exposure to UV and heat.

### 2.2. Structural Analysis

**Figure 6:**
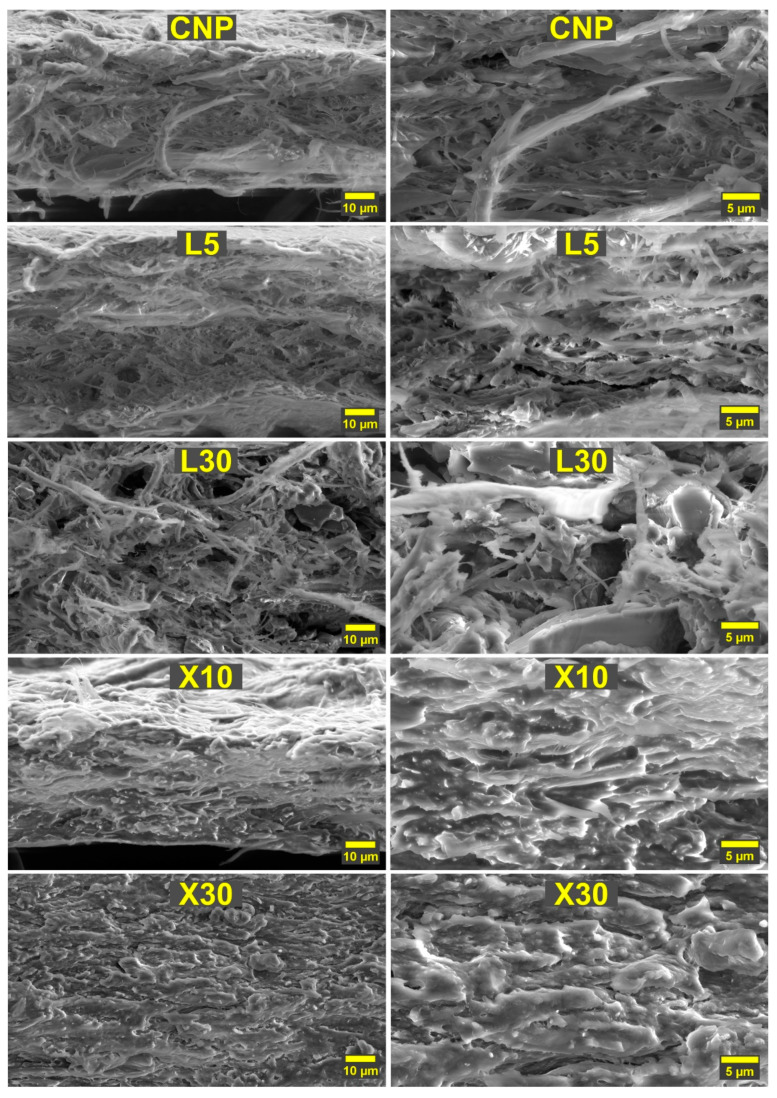
Cross-section scanning electron micrographs of selected NP compositions.

Figure 6 shows a typical scanning electron micrograph of selected NP compositions. SEM images revealed closely packed cellulose fibrils with an interconnected network that showed visible porosity and no structural deformations. The introduction of lignin in L5 NP shows that a small layer of lignin nanoparticles has been accumulated on the surface of cellulose fibrils and in the previously visible pores. The lignin addition has contributed to visible layer delamination and the formation of microscale voids. This explains the decreased mechanical performance observed for lignin NP. The L30 NP SEM images reveal a strongly disrupted cellulose fibril network with a chaotic structure that includes large voids. In high loadings, lignin appears to thoroughly coat cellulose fibrils, contributing to a significant decrease in hydrogen bonding between fibers and a subsequent drop in mechanical properties. While the lignin NP structure revealed the inherent issue of compatibility between components, it also yields unique changes in structure. These voids could be used to deliver biologically active components for wound treatment [48].

In contrast, the addition of xylan to CNP showed a densely packed structure with no visible pores or voids in the structure of X10. Our observations coincide with the literature, where xylan shows the ability to fill gaps between cellulose fibers and improve properties in low loadings [49,50]. The smaller molecular weight and more branched structure contribute to xylan’s ability to insert itself into gaps between cellulose fibers and coat them. In addition, xylan effectively enhances the hydrogen bond network of cellulose; thus, there is no visible separation between xylan and cellulose in X10 NP. At the same time, X30 NP shows clear phase separation, and xylan forms visible layers of interphase. Individual cellulose fibrils are almost indistinguishable, while the highly dense structure remains. Thus, thick interphase layer formation results in poor mechanical performance due to disrupted load transfer in the cellulose network [51,52]. Xylan’s ability to form dense structures with no visible pores could be used to improve gas barrier properties either by addition or through coating means [38].

**Figure 7:**
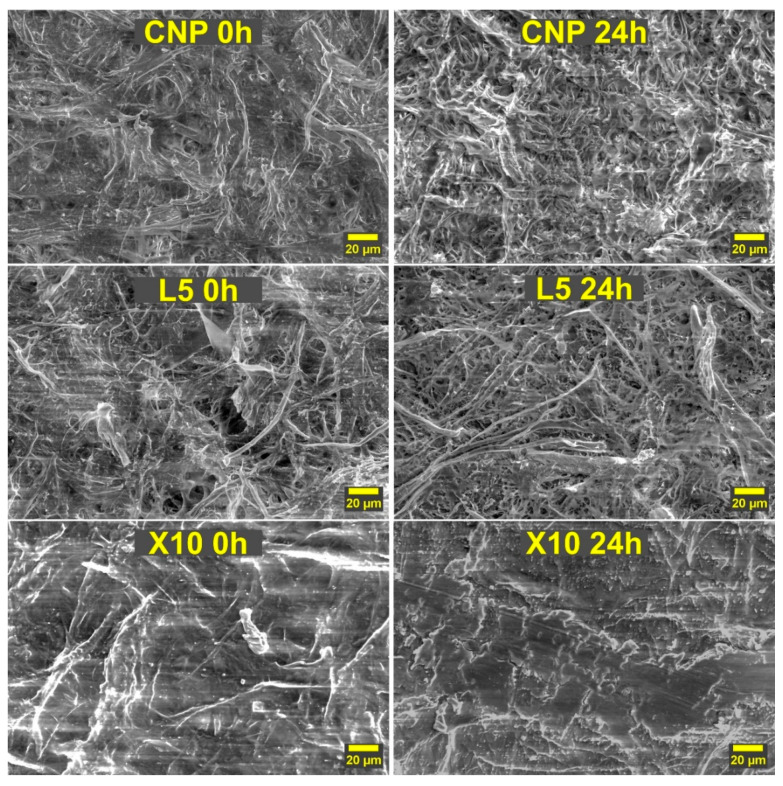
The impact of 24 h of UV irradiation on the surface morphologies of selected NP compositions.

The surface morphologies of unaged and aged NP have been studied by SEM (Figure 7). The 24 h irradiation and heat treatment were chosen to maximize differences between the compositions. The unaged NP surface yields similar observations to those previously discussed above. Before aging, all NPs showed a relatively smooth surface with interconnected cellulose fibers. After aging, the CNP surface becomes very rough and individual fibers become more pronounced, which indicates that small fibrils connecting large fiber formations have degraded. The loss of interconnected fiber networks is reflected in the observed catastrophic failure of mechanical performance properties. The higher magnification images provided in the Supplementary Materials (Figures S1 and S2) reveal intensive cracking of cellulose fibers. Cracking indicates the breaking of (1→4) β-glycosidic bonds, effectively reducing the molecular weight of cellulose [53,54]. In addition, cracks serve as stress concentrations that contribute to low tensile properties after UV aging.

The L5 NP after aging shows a rather interesting change with the introduction of small holes throughout the surface. The interconnected network of cellulose fibers is severely damaged, but it remains in better condition than CNP. Lignin particles could coat cellulose fibers and significantly reduce the cracking due to efficient irradiation absorption as indicated by higher magnifications in the Supplementary Materials (Figures S1 and S2). Overall, the L5 composition follows a similar route to CNP, with the drastic increase in surface roughness and separation of individual fibers. Lignin could not last a full 24 h and, as indicated by tensile tests, it seemed to fail between 12 and 24 h. In the case of xylan NP, it can be observed that the surface remained relatively smooth, with the top layer experiencing significant degradation, as revealed by no visible fibers in the aged surface. This could be explained by cellulose and xylan degradation products remaining on the surface and forming a protective layer.

The depth of UV irradiation damage can be seen in Figure S3. CNP had almost complete penetration with UV, significantly damaging the cellulose fiber network, which was seen as a loss of dense structure. The L5 crosscut showed a much lower penetration depth compared to CNP. While the smooth surface of the L5 composition has degraded, the damage has been contained close to the surface. Similarly, the X10 composition preserved the structural integrity of CNP and limited UV structural damage to the surface of NP. The surface of X10 has disrupted the structure, and there is a visible difference seen as an almost different layer for the exposed surface.

### 2.3. UV-VIS and FTIR Spectroscopy

As shown in Figure 5, UV-VIS spectroscopy takes advantage of the induced color changes in NPs after aging them in UV irradiation and heat. The presence of oxidizable groups results in color change [55,56]. Visual changes show CNP changed color from white to white with a yellow tint, brown lignin NPs experienced whitening, and xylan NPs changed from yellow to a light brown color. This loss of color (whitening) for cellulose materials has been reported in the literature and indicates lignin degradation [23,57,58]. The yellow tint observed for CNP indicated an oxidation of cellulose [59,60].

**Figure 8:**
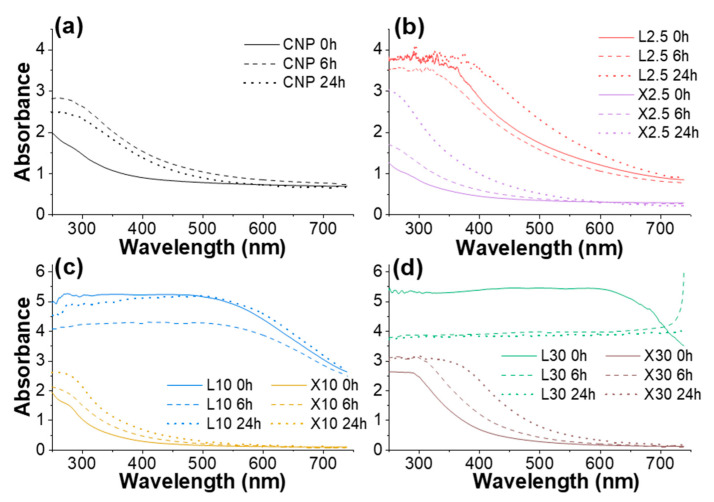
UV-vis spectra of NPs before and after aging 6 h and 24 h: (**a**) CNP, (**b**) L2.5 and X2.5, (**c**) L10 and X10, (**d**) L30 and X30.

The absorbance spectra of tested NPs are shown in Figure 8. The spectra are relatively simple and have absorbance at around 280 nm for all compositions and an extended absorbance up to 600 nm for lignin NPs. The absorbance from 250 to about 350 nm is relatively weak for Xylan NP and CNP before UV exposure. No absorption peaks in visible light are seen for CNP and xylan NP, which indicate a lack of chromophore groups in xylan and cellulose structures. After aging, there is a visible shift to around 300 nm and an increase in absorption intensity. This is attributed to the formation of various cellulose and xylan oxidation products, such as carbonyl, aldehyde, and carboxyl groups [43]. This is observed as a loss of whiteness for these NPs.

UV-vis spectra indicate the increase in lignin concentration. Lignin is known for its aromatic structure that contains mainly conjugated double bonds, which show very high absorbance in UV-vis spectra compared to non-conjugated double bonds [24]. Lignin NP spectra (L10 and L30) show a decrease in absorption intensity, which is the opposite of observations for CNP and xylan NP. This is commonly explained as a breakdown of the lignin structure (formation of low molecular weight compounds) and results in a decrease in conjugated systems [61]. Lignin acts as a barrier to the UV light that protects cellulose in natural structures such as wood and plants [21]. Protection from UV is achieved by the absorption of light and degradation of lignin’s structure.

In the case of L2.5, UV degradation results are comparable to CNP, indicating that lignin cannot effectively shield UV rays from cellulose fibers at this concentration due to the non-homogeneous coating of cellulose fibers. The absorption of xylan NPs drops significantly above 350 to 400 nm when compared to CNP. This confirms that xylan effectively coats the cellulose fibers’ surfaces, thus reducing the absorption of visible light.

**Figure 9:**
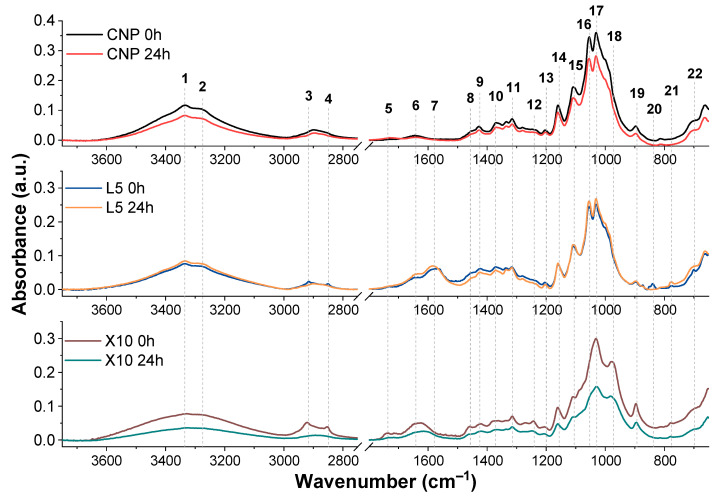
FTIR spectra of CNP, L5, and X10 samples before and after 24 h UV irradiation. The FTIR region between 1800 and 2800 cm^−1^ is emitted because it lacks any significant bands.

Figure 9 show FTIR spectra of NP samples before and after 24 h of UV irradiation, while Table 1 summarizes the absorption band assignments. L5 and X10 samples were selected for the FTIR analysis due to their mechanical property preservation compared to CNP. The spectra for CNP show the highest absorption at 1000 to 1100 cm^−1^ region, indicating characteristic cellulose peaks for C-O linkages. The addition of lignin reduces the intensity of these peaks, indicating partial surface coating. At the same time, lignin aromatic ring C = C double bond stretching (7) is introduced in the L5 spectra [62]. In contrast, adding xylan alters the characteristic double C-O peak intensities and introduces a new C-O peak at 976 cm^−1^, which is commonly used for xylan identification [63]. Both modifiers influenced the -OH group absorption, further proving changes in surface structure and hydrogen bond formation.

After 24 h of aging, CNP showed a significant decline in tensile properties, which is reflected in the decreased absorbance of C-O and C-O-C groups. Cellulose mechanical properties are mainly determined by the ability to form hydrogen bonds and molecular weight [64]. As C-O-C cleavage occurs, the molecular weight of cellulose is reduced, while the reduction in C-O absorbance could be attributed to surface oxidation. In addition, a new peak (5) representing C = O is observed in the CNP spectra, testifying to the surface oxidation process.

In the L5 composition, the absorbance of characteristic cellulose peaks increases after aging. This could be attributed to the breakdown of lignin, which coated the surface of cellulose fibers and is supported by the shift observed for C = C peak absorbance (7) and matches the data from the literature, where lignin degradation leads to the breakdown of the structure and conjugated double bond systems [61]. Thus, a shift to lower conjugation of double bonds is seen as a shift in the absorbance peak. The loss of the characteristic lignin peak at 837 cm^−1^ on the surface further proves lignin degradation.

The absorbance intensity for X10 NP almost halved after 24 h of UV irradiation, indicating significant structural degradation. Although SEM analysis (Figure 6) shows that xylan effectively coats cellulose fibers, it has been reported in the literature that xylan has poor UV resistance [65]. There are no visible indications of FTIR spectra changing to match with cellulose, indicating that degradation is partial and degraded xylan remains on the surface of NP. Thus, while xylan did significantly degrade, the rigid cellulose fibers were mainly protected, as indicated by the tensile properties. The FTIR observations match the visual changes, where CNP and xylan NPs showed visible signs of oxidation while lignin degraded and lost its distinct brown color. Similarly, xylan samples had the highest decrease in absorbance spectra and the most pronounced color shift after UV aging.

**Table 1:** Assignments of the infrared absorption bands.

Band	Wavenumber (cm^−1^)	Assignment	References
1	3333	Intramolecular hydrogen bonding of -OH group	[66]
2	3276	Intermolecular hydrogen bonding -OH group	[66]
3; 4	2915, 2850	CH symmetrical and asymmetrical stretching	[66]
5	1738	C = O stretching of acetyl or carboxylic acid in hemicellulose	[23]
6	1640	C = O stretching in the carboxyl group	[67]
7	1582–1560	C = C stretching of lignin aromatic ring	[62]
8	1458	CH bending of xylan	[63]
9	1427	CH_2_ scissoring	[66]
10	1372	C-H bending	[66]
11, 22	1315, ≈700	CH_2_ rocking	[43,66]
12, 13, 15, 16, 17	1242, 1202, 1110, 1055, 1030	C-O stretching	[63,66,67]
14	1160	C-O-C asymmetric bridge	[23]
18	976	C-O stretching in xylan	[63]
19	895	β-linkage of cellulose	[66]
20, 21	837, 776	C-H out of plane deformation in lignin aromatic ring	[63,68]

### 2.4. Moisture Effect on the Tensile Properties

The hydrophilic nature of cellulose-based materials results in properties that are highly sensitive to humidity variations. This should be considered when evaluating their performance properties. The equilibrium moisture contents w_∞_ for all measured compositions are listed in Table 2. The amount of absorbed moisture in CNP and modified NP films significantly increases with RH. For CNP, the increase in w_∞_ is about 26% (from 2 to 28%) for samples conditioned at RH24% and RH97%, respectively. The moisture absorption capacity of lignin-modified NPs is significantly higher: w_∞_ of L5 and L10 samples conditioned under RH97% exceeds 70%. Xylan NP compositions demonstrate up to a 40% weight increase, which is almost two-fold lower than w_∞_ for lignin-modified NPs.

Higher lignin concentration promoted moisture absorption, but in the case of xylan, the lowest concentration (X1) showed the highest absorption values. The lack of synergy between cellulose and lignin particles resulted in agglomeration and phase segregation, which could explain the high moisture absorption for lignin NP compositions. Österberg et al. described the importance of particle shape and size for lignin composites to achieve good packing, mechanical, and thermal properties [69]. This could explain high water absorption as the lignin phase could induce defects and cavities in the cellulose fiber mesh network by increasing the voids and the surface that interacts and absorbs moisture. Xylan is a more branched molecule than cellulose. Thus, more side chains contain many more OH groups that can form hydrogen bonds with cellulose and water.

In addition, hemicellulose (xylan) tends to fill gaps between cellulose fibrils, thus creating a denser interface coating under high loadings up to 10 wt% [44]. In this regard, the low xylan concentration in the X1 and X2.5 samples contributes to significant enhancement of the NP interface, but unlike higher loadings, it cannot pack fibrils in such a dense manner. This is seen in the RH 75% and RH97% results, where X5 and X10 NPs are within the margin of error, but X1 has a significantly higher relative weight gain.

Samples with filler loadings of 20 wt% and 30 wt% showed a significant increase in water absorption capacity due to the disruption of the cellulose fiber mesh network, which dramatically affected the mechanical properties, and thus, they were not further analyzed. The higher moisture absorption capacity of lignin-modified nanopaper resulted in a more significant hygrothermal impact on the mechanical properties than CNP and xylan NP.

**Figure 10:**
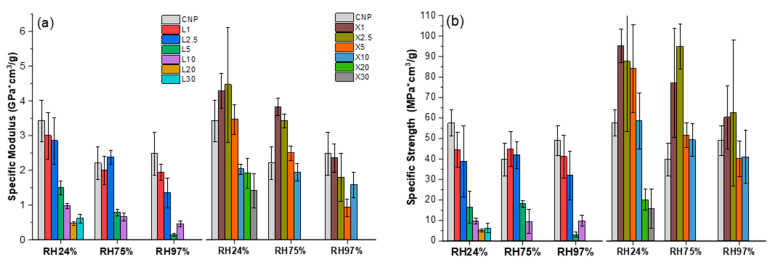

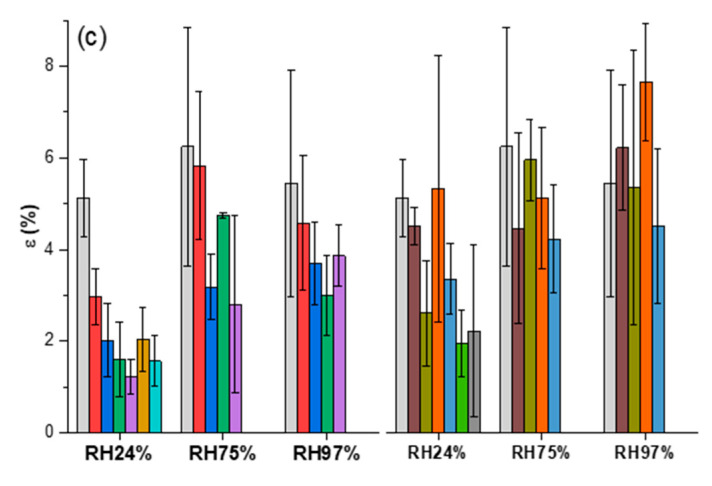
Tensile properties: (**a**) specific elastic modulus, (**b**) specific ultimate tensile strength, and (**c**) elongation at break for NPs saturated under different RH.

Tensile tests were conducted after sample weight was stabilized under three selected RH values: 24%, 75%, and 97%. A comparison of tensile properties in different RHs for NPs is presented in Figure 10. Moisture absorption greatly affected the mechanical behavior of the NPs, resulting in a decrease in the elastic and strength characteristics with higher water saturation. Lignin-modified NPs are characterized by lower tensile properties and their higher sensitiveness to moisture than the CNP. This results in a complete loss of operation properties of highly loaded lignin NPs and makes these compositions almost unusable at high RH. The addition of xylan, on the contrary, resulted in the improvement of the mechanical characteristics of CNP.

Specific elastic modulus values increase by 32% and 55% for X2.5 NP compared to CNP at RH24% and RH75%, respectively. Absorbed moisture plasticized the material, although the elastic modulus and strength of moisture-saturated (RH 97%) xylan NPs is comparable to CNP. For lignin NPs, a decrease in the elastic modulus and tensile strength was observed. Correspondingly, Xylan NPs showed a remarkable 2.3-fold increase in specific tensile strength at RH 75% for X2.5 composition. Tensile strength was comparable to CNP at RH97%, similarly to changes observed for elastic modulus. Elongation values showed an opposing trend and increased with moisture content. Lignin NPs still had values that were lower than CNP, but xylan NPs showed comparable values. All strain measurements showed relatively large data scatter, as indicated by error bars.

The hydrophilic nature of cellulose results in strong interactions with water molecules. This interaction yields strong plasticizing and swelling effects on cellulose-based structures [70,71,72]. As a result, intermolecular bonds between cellulose and cellulose or fillers are reduced and replaced with hydrogen bonds formed with water molecules. Plasticizing leads to reduced stiffness (elastic modulus) and decreased intermolecular bonds between components, lowering the tensile strength. However, in the case of elongation, slippage between fibers is improved, yielding higher strain values [73]. High moisture content promotes swelling, and as a result, the volume of NPs is affected by the internal expansion that results in a significant decrease in all tensile (mechanical) properties. This was observed for samples L5 and L10 at RH97%. Cazón et al. also reported that bacterial cellulose films showed a strong dependence on moisture content [74]. According to the authors, Guo et al. studied four types of nanocellulose and their equilibrium moisture content, which can significantly vary and depend on cellulose structure and crystallinity [72].

**Figure 11:**
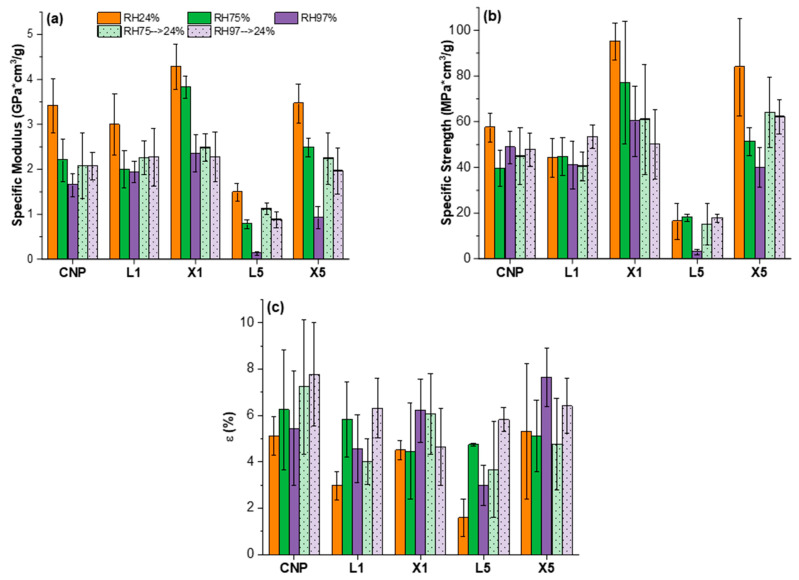
Tensile properties: (**a**) specific elastic modulus, (**b**) specific ultimate tensile strength, and (**c**) elongation at break of CNP, L1, L5, X1, and X5 NPs at different RH (24%, 75%, 97%) and their properties retention after moisture desorption.

Decreased properties can be expected with higher moisture content for hydrophilic materials, but the ability to recover initial properties must be studied. After the removal of excess water, it is essential to understand changes in durability. Figure 11 shows the selected composition’s ability to recover mechanical properties after water desorption. In this case, samples exposed to RH75% and RH97% were conditioned back to RH24%. Absorbed moisture results in irreversible structural changes in all compositions. Retention of the elastic modulus is in the range of 60%, 75%, and 60% for CNP, lignin NPs, and xylan NPs, respectively. The tensile strength of the compositions was retained to a greater extent, around 80%, 95%, and 70%. Variations of the strain at failure for all samples were in the data scatter range. It is interesting to note that despite the significant difference in moisture saturation levels at RH75% and RH97% (Table 2), irreversible impacts after moisture desorption are similar for both groups of samples (the relative retention is almost the same for RH75 (>24% and RH97 (>24%)). The loss of tensile properties can be explained by defects induced by sample swelling that leave various voids and structural defects after the desorption of excess water. Water migration has been shown to have a negative impact on cellulose reinforcement in composite structures when exposed to artificial weathering [75]. As is discussed by Dufresne in his review of cellulose-based material applications, extensive exposure to water should be avoided to prevent the degradation of nanostructure [76]. High moisture content increased tensile properties in hot-pressed cellulose films, according to Liu et al. [40], because the structure was relieved of internal stresses. Tensile characteristics of bacterial cellulose films drop by up to 50% and 75% for tensile strength and elastic modulus, respectively, when RH50% was elevated to RH75% [77].

### 2.5. Nanopaper Performance Quality Analysis

**Figure 12:**
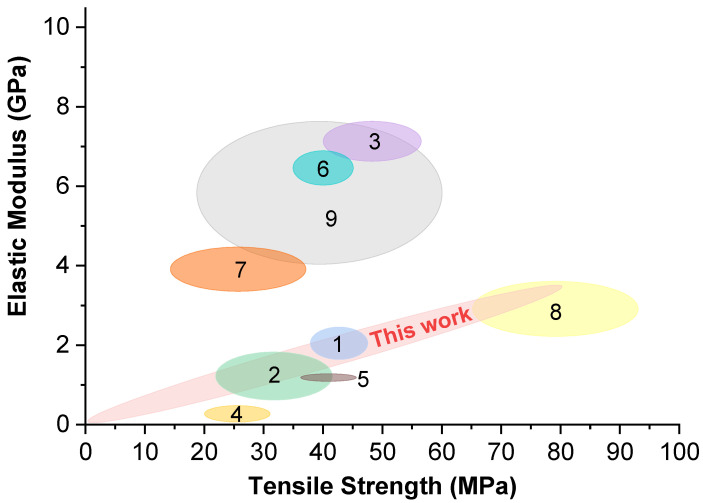
Elastic modulus and tensile strength map to compare CNPs prepared by similar studies from the literature. (1) MFC/lignin/xylan film [40]. (2) Microcellulose/hydrolysed lignin/xylan film [50]. (3) Carboxymethylated NFC/Xylan NP [51]. (4) Xylan/carboxymethyl cellulose (CMC), oxidized carboxymethyl cellulose (OCMC) and cellulose nanofibers (CNFs) film [33]. (5) Bacterial cellulose nanofibrils and nanocrystals film [77]. (6) Bleached and unbleached pistachio shell MFC film [78]. (7) Cellulose nanowhiskers (CNW) and NFC nanopapers [79]. (8) Acetylated rye arabinoxylan (AcAX)/NFC films [80]. (9) Sugarcane bagasse NFC films [81].

There is a large discrepancy in the tensile properties of NPs in the literature. The NPs’ mechanical performance is characterized by Figure 12, which summarizes the tensile properties of NPs from various studies. Hasen et al. (3) demonstrated a very high elastic modulus, reaching 7.6 GPa for carboxymethylated NFC/Xylan NP prepared 50/50 (*w*/*w*) [51]. Pistachio shells (6) were mildly treated to process them into dimensions closer to microfibrillated cellulose than NFC [78]. The authors obtained relatively high elastic modulus, but the dimensions of cellulose contributed to lower stress and elongation values. Xiong et al. used a combination of cellulose nanowhiskers (CNW) and NFC to produce NP (7) [79]. Rigid rod-like CNW provided high elastic modulus and yielded relatively brittle NP, which could be compensated for with the addition of NFC but at a loss of elastic modulus. In general, xylan NPs demonstrate poor mechanical performance, but, at the same time, acetylated arabinoxylan films (8) can achieve high properties comparable to cellulose NPs [80]. Tedeschi et al. combined hydrolyzed lignin with xylan and microcellulose compositions using a solvent system to cast bioplastics [50]. As seen by the properties of films (2), using solvents does not guarantee higher properties than conventional casting from suspensions. Due to the lack of density values and chemical composition data in some studies, it is hard to compare various NPs directly. For accurate comparison, specific elastic modulus and strength values would be preferable, as well as a similar level of room humidity.

The NPs produced in this study show a wide range of mechanical properties. As seen from the literature data, NP properties can be improved with chemical modification, by changing the chemical composition of NFC (the amount of lignin and hemicellulose) and by combining NFC with different types of fillers. In contrast, the proposed NCs’ interface engineering using lignin and xylan additives could provide a sustainable and straightforward route to control the mechanical properties. The higher quality of NFC provides more significant tensile strength (stress) and elongation values but has decreased elastic modulus. At the same time, the elastic modulus is improved by the addition of smaller, more rigid particles or reduced chemical treatment of the cellulose source, but these NPs can be brittle. As our source cellulose is already highly purified and, thus, has a damaged structure from chemical pretreatment, lower elastic modulus values can be expected.

**Figure 13:**
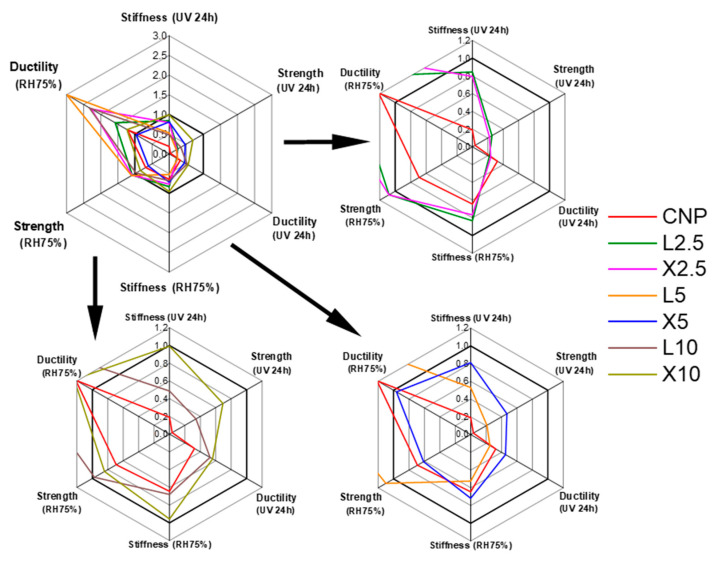
Relative changes of performance properties for NPs before and after aging. For reference, dry samples were used for UV and samples saturated at RH24% were used for water adsorption impacts. Value 1 represents the sample before aging.

As shown in Figure 13, a radial plot displays NP performance multivariate observations with the most desirable variables of durability characteristics—stiffness (modulus), strength, and ductility (elongation) at 24 h of UV light and RH75% aging. The relative changes in tensile properties for modified NPs before and after aging are shown, where UV irradiated dry samples and samples saturated at RH24% were chosen as the reference. The unaged NP represents value 1 on the plot. The performance quality of the NP was enhanced after cellulose interface modification with the bio-based additives of xylan and lignin.

The impact of UV light irradiation is more critical to the CNP, resulting in a substantial decrease in mechanical properties. The chosen bio-based modifiers significantly enhanced the mechanical performance. Both modification methods provide enhanced moisture resistance with low loadings of 2.5 wt%. Unfortunately, low loadings did not demonstrate significant UV resistance. Xylan’s impact on the strength, stiffness, and ductility (UV 24 h) properties was superior to lignin’s. The xylan modification of the cellulose interface with 10 wt% showed the overall best performance. In contrast, the lignin modifier shows almost a three-fold improvement in the NP’s ductility (RH75%) and provides good moisture resistance at low loadings. Considering the food packaging application, the optimal NP samples are X10 and L2.5, which present the highest performance characteristics considering the durability in the UV-light and humidity aging tests.

## 3. Materials and Methods

### 3.1. Materials

High-purity cellulose was obtained from old unutilized laboratory filter paper. Paper was shredded using Retsch SM300, with a sieve size of 2.00 mm. The process was repeated with a sieve size of 0.25 mm, and for both pass-throughs, the mill rotation speed was 1500 rpm. Laboratory-grade sodium hydroxide, beechwood xylan, and k raft lignin were purchased from Merck KGaA (Darmstadt, Germany) and used as received without additional processing.

### 3.2. Cellulose Nanofibril Preparation

**Figure 14:**
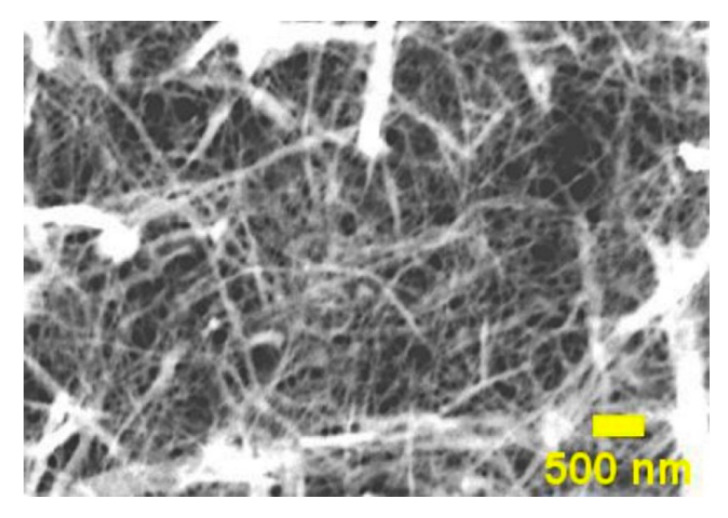
STEM image of NFC.

A suspension of 1 wt% nanofibrillated cellulose (NFC) was prepared in deionized water (DI). The obtained aqueous suspension was mixed in an ordinary kitchen blender (800 W, 5 min) and then homogenized with the high shear mixer L5M-A (Silverson Machines LTD, Chesham, UK) for 3 min (5500 rpm). After homogenization, it was processed with a microfluidizer (LM20, Microfluidic, Westwood, MA, USA) equipped with an H210Z (200 µm) chamber. Even defibrillation was achieved using 5 cycles. The microfluidizer pressure was set to 30,000 psi. Scanning transmission electron microscope (STEM) FEI Nova NanoSEM 650 Schottky field emission scanning electron microscope (FESEM) was used at acceleration voltage 10 kV to obtain an image of NFC suspension. The sample was prepared from a diluted NFC suspension droplet, which was placed on a copper grid, and water was allowed to evaporate; the obtained image is shown in Figure 14.

### 3.3. Nanopaper Preparation

Kraft lignin was suspended in DI water and stirred magnetically at 85 °C for 1 h. A strong alkaline solution of NaOH was slowly added to stabilize pH at 10. The final concentration of suspension was adjusted to 10 wt% lignin. Similarly, xylan was dissolved in DI and stirred magnetically at 85 °C for 1 h. After preparation, suspension and solution were both cooled to room temperature (20 °C).

The 1 wt% NFC suspension was combined with previously prepared lignin suspension or xylan solution using a blender (800 W, 5 min), and further homogenization was achieved with magnetic stirring for 2 h at room temperature (20 °C). Polystyrene (PS) Petri dishes were used for film casting and drying at room temperature (20 °C). Dry films were further dried in a thermostat at 50 °C for 24 h. Modified CNPs were prepared with the following concentrations of lignin or xylan: 1, 2.5, 5, 10, 20, 30 wt%. Nanopaper from pure NFC with no modifiers, i.e., cellulose nanopaper (CNP), was also produced for comparison. Prepared NPs were abbreviated, combining letters from lignin (L) or xylan (X) with their loading in wt% (e.g., X1 sample corresponds to NP with 1 wt% xylan and 99 wt% NFC loadings, L1 sample—lignin 1 wt% and NFC 99 wt%). Densities of prepared NPs are listed in Table 3. Samples with thicknesses around 0.1 mm in the shape of strips, 10 mm in width and 40 mm in length, were cut from films. Before testing, samples were stored in a thermostat at 50 °C and RH < 10%. These samples are considered dry reference samples.

### 3.4. UV Irradiation and Tensile Tests

The samples were removed from the thermostat (50 °C and RH < 10%). Films were irradiated with 1.6 W/cm^2^ intensity and at a fixed distance of 25 cm from the source. A deep UV exposure lamp (Hg, 1000 W) with a broad emission spectral range from 200 to 600 nm was used as an irradiation source in an air environment. The constant temperature of 80 °C was maintained in the experimental chamber with the UV lamp. Exposure time was set to 6, 12, and 24 h. After irradiation, samples were collected in sealable plastic bags and kept for 24 h before performing a tensile test.

A Tinius Olsen model 25ST (USA) universal testing machine was set up with a load cell of 5 kN. The crosshead speed was set to 1 mm/min, and the gauge length was 20 mm. Five parallel measurements for each NP sample were performed at room temperature (20 °C) and ambient conditions (RH40%).

### 3.5. Spectroscopy

UV-vis absorbance was measured in a range from 240 to 740 nm using a SolidSpec3700 UV-VIS-NIR Shimadzu (Kyoto, Japan) spectrophotometer. A white BaSO_4_ plate was used as the reference plate for all measurements. Three parallel measurements were combined in the final spectra.

Fourier transform infrared spectroscopy (FTIR) spectra of NP samples acquired in attenuated total reflectance mode were recorded on a Nicolet 6700. (ThermoScientific, Karlsruhe, Germany). FTIR spectra were recorded at a resolution of 4 cm^−1^ in the 400–4000 cm^−1^ range. Sixteen measurements were taken on each specimen, and the average spectrum is given.

### 3.6. Moisture Absorption and Tensile Tests

Samples were conditioned in desiccators under different relative humidity environments (RH%) at room temperature 22 °C. The humid environments were created by using different saturated salt solutions: KC_2_H_3_O_2_ (RH24%), NaCl (RH75%), and K_2_SO_4_ (RH97%). Gravimetric measurements were made with an accuracy of 0.01 mg, and the relative weight changes of samples w [%] were determined as weight gain per weight unit. Moisture saturation was achieved within 3–7 days. Retention of the mechanical properties after moisture desorption was studied on samples initially saturated at RH75% and RH97% and then conditioned at RH24% until weight stabilization, which was abbreviated as RH75 (>24%) and RH97 (>24%), respectively.

The tensile properties of moisture-saturated samples were tested using a Zwick testing machine with a load cell of 2.5 kN at a crosshead speed of 1 mm/min. Tabs from the paper tape were applied to the samples, and the gauge length was set to 20 mm. The elastic modulus was determined in the linear part of the stress–strain curve within the strain range of 0.2–0.5%. Five replicate samples were tested immediately after their extraction from a desiccator (within 1 to 2 min) for each NP composition and RH.

### 3.7. Structural Analysis

The microstructure of the surface and cross-section of the NPs were observed with FEI Nova NanoSEM 650 Schottky field-emission scanning electron microscope (FESEM) at an acceleration voltage of 10 kV. The films were frozen in liquid nitrogen for 2 min before testing and then broken.

## 4. Conclusions

The proposed cellulose nanopapers modification, using lignin and xylan additives, provides simple and sustainable route to engineer the interface in the developed mesh material and control the materials durability to the UV irradiation, moisture, and temperature. The tensile properties are significantly enhanced with the addition of xylan and lignin interface modifiers. The UV irradiation showed a milder impact on the cellulose modifications with 5 and 10 wt% xylan concentrations. In comparison, the moisture absorption test showed the overall best performance for xylan’s 2.5 wt% composition. Xylan interface development in all sample concentrations showed remarkable improvements in the UV and heat resistance of NP. Lignin also strongly improved the UV resistance of NP. Cellulose NP without interface modifiers exhibited a catastrophic decrease in tensile properties after UV exposure. The UV irradiation damage is revealed in SEM analysis.

The oxidation and depolymerization of lignin and xylan at the protective interface for the cellulose was indicated by UV-Vis spectroscopy as a shift in absorption peak intensities and regions. Similar observations were made from FTIR spectra analysis. The moisture sorption capacity of the NP increases with moisture level and with lignin and xylan loadings. High moisture content promoted NP swelling, which failed samples with 20 and 30 wt% loadings of lignin and xylan. Absorbed moisture significantly affects the elastic modulus and strength characteristics of CNP, but resistance was improved for modified NP. The lignin modifier resulted in higher moisture absorption capacity and, as a result, higher properties’ sensitivity to humidity changes due to the strong agglomeration and phase segregation effect. However, remarkably, lignin NP showed better property retention even at high moisture content compared to CNP. The retention of the elastic modulus and strength after moisture desorption is in the range of 60–95%; lignin-modified compositions show the highest property retention.

The NP films of X10 and L2.5 have presented the highest durability performance characteristics against UV-irradiation and humidity aging. The improvements were achieved by improving the interface (xylan), while the developed interphase layer covered the cellulose mesh. These completely sustainable NP film compositions are being considered for the food packaging application, which is now underway as validation for berries, fruits, and vegetables.

## Figures and Tables

**Table 2:** The moisture content at saturation for NPs measured at different RH.

Sample	w_∞_, %		
	RH24%	RH75%	RH97%
CNP	2.1 ± 0.5	7.1 ± 0.6	28.0 ± 3.0
L1	2.2 ± 0.3	8.7 ± 0.4	55.6 ± 5.5
L5	2.3 ± 0.1	13.6 ± 2.0	73.8 ± 4.1
L10	2.9 ± 0.5	16.0 ± 3.5	76.6 ± 4.0
L20	2.5 ± 0.2	-	-
L30	2.6 ± 0.4	-	-
X1	2.2 ± 0.1	19.6 ± 1.0	42.6 ± 5.1
X5	2.3 ± 0.2	11.4 ± 0.7	31.7 ± 4.3
X10	2.5 ± 0.2	12.2 ± 1.2	34.4 ± 4.0
X20	2.4 ± 0.2	-	-
X30	2.3 ± 0.2	-	-

**Table 3:** Densities of prepared NPs.

Sample	Density (g/cm^3^)	Standard Deviation (g/cm^3^)
CNP	0.82	0.10
L1	0.54	0.05
L2.5	0.62	0.05
L5	0.67	0.11
L10	0.76	0.07
L20	0.81	0.16
L30	0.54	0.02
X1	0.63	0.05
X2.5	0.76	0.05
X5	1.07	0.20
X10	1.17	0.10
X20	1.38	0.07
X30	1.44	0.08

## Data Availability

The data presented in this study are available in Supplementary Material.

## Supplementary Materials

The following are available online at https://www.mdpi.com/article/10.3390/ijms222312939/s1.
