# Supplementary material for: Lignin and Xylan as Interface Engineering Additives for Improved Environmental Durability of Sustainable Cellulose Nanopapers
Source: Int J Mol Sci. 2021 Nov 29;22(23):12939. doi: 10.3390/ijms222312939 (PMC8657447; doi:10.3390/ijms222312939)
Supplement: Supplementary file 1 [file ijms-22-12939-s001.zip › ijms-1466442-supplementary.pdf]

Supplementary

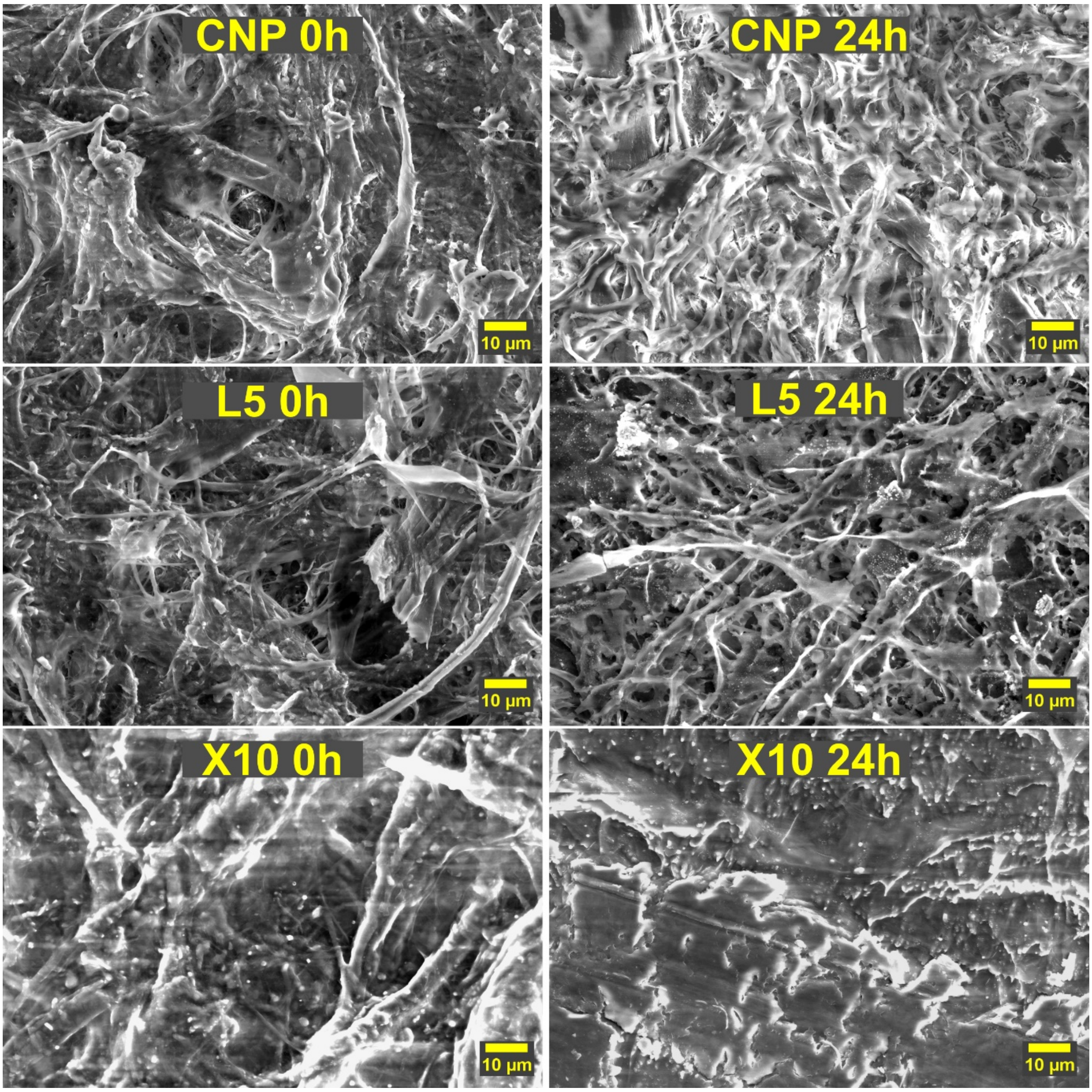

Figure S1. SEM micrographs showing the surface of selected NP compositions before and after UV aging, at a magnification of 1000x.

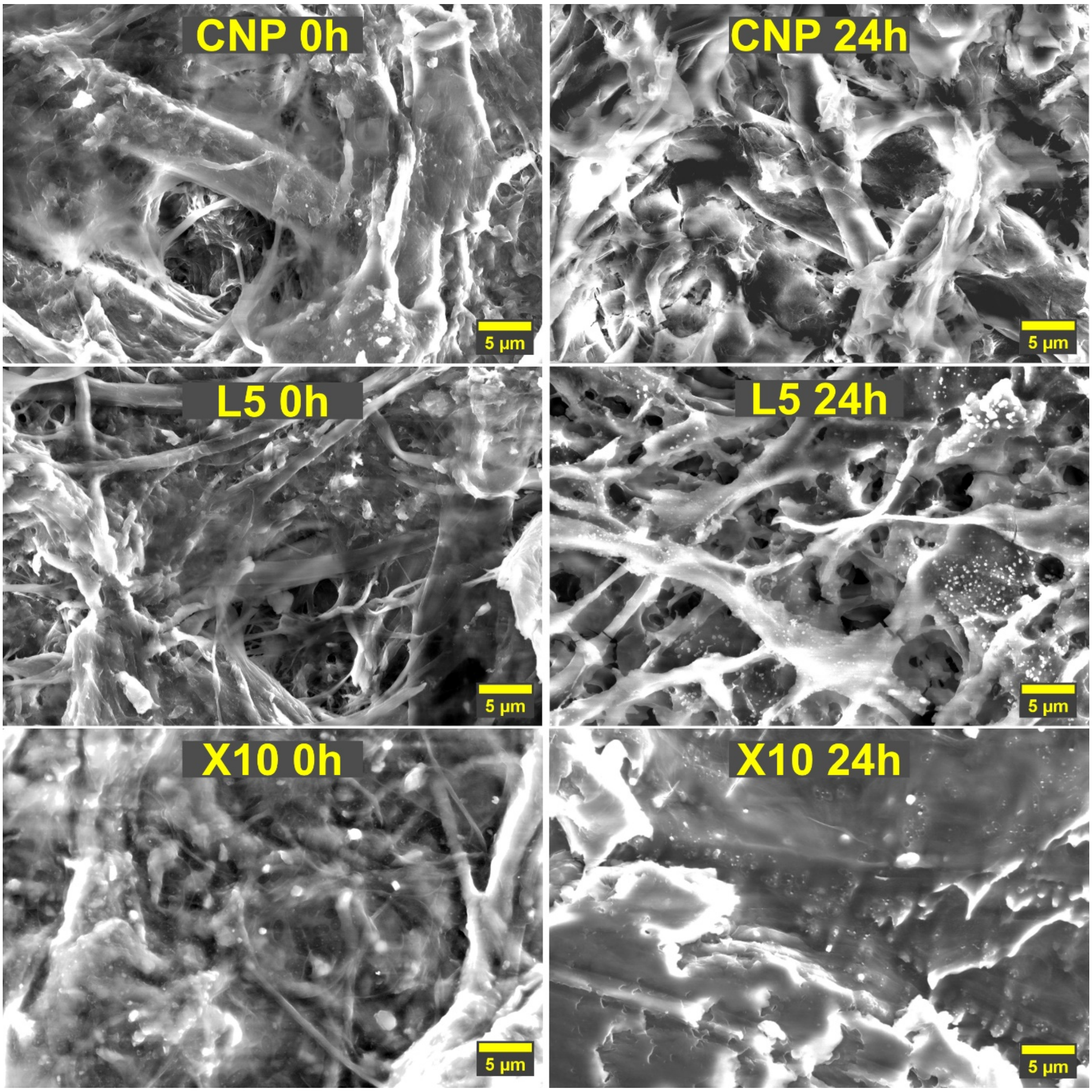

Figure S2. SEM micrographs showing the surface of selected NP compositions before and after UV aging, at a magnification of 2500x.

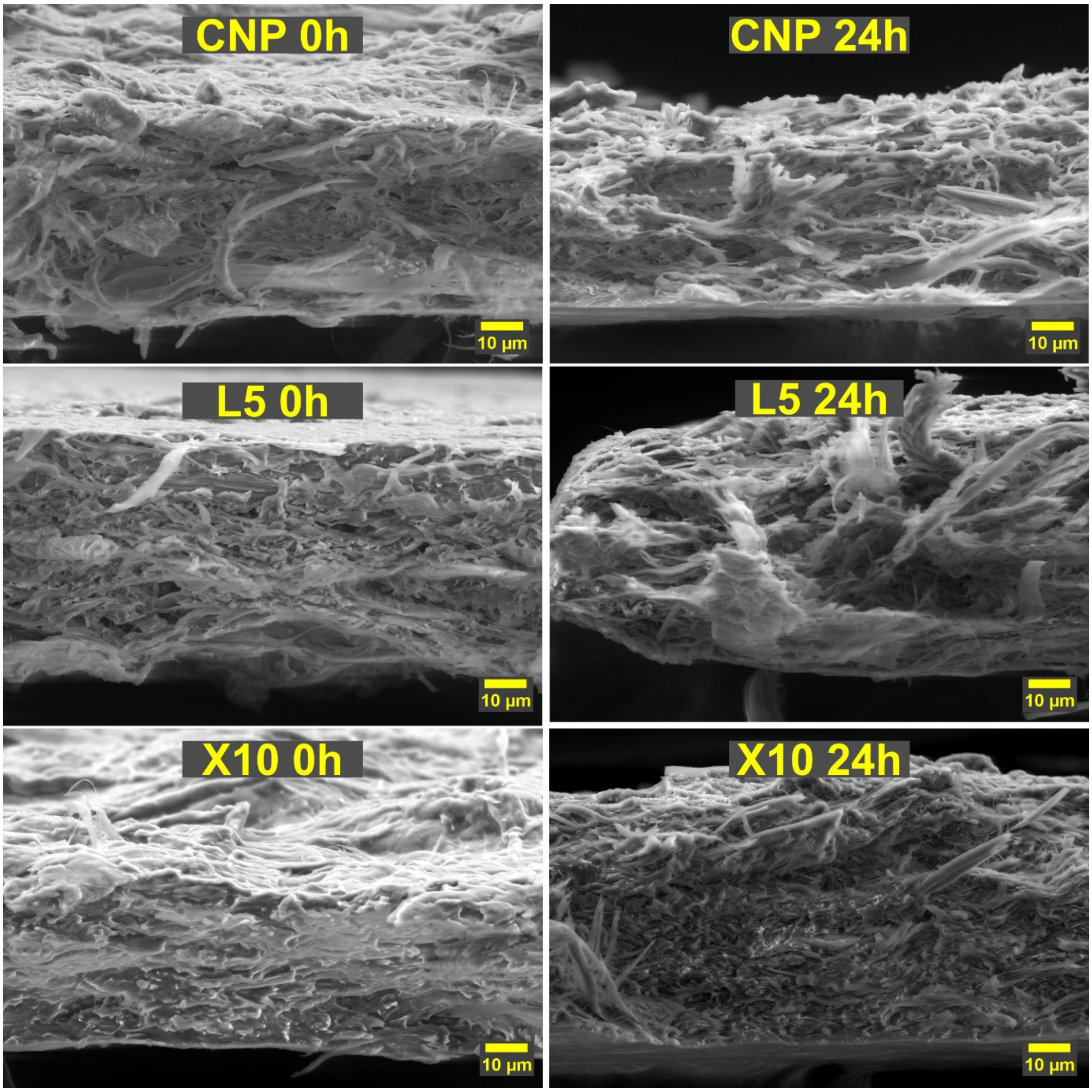

Figure S3. SEM micrographs showing the cross-section of selected NP compositions before and after UV aging, at a magnification of 1000x.
